# The relationship between functional health literacy and the use of the health system by diabetics in Switzerland

**DOI:** 10.1093/eurpub/ckt202

**Published:** 2013-12-22

**Authors:** Jasmin Franzen, Sarah Mantwill, Roland Rapold, Peter J. Schulz

**Affiliations:** 1 Helsana Insurance Company Ltd, Zurich, Switzerland; 2 Institute of Communication & Health, University of Lugano, 6900 Lugano, Switzerland

## Abstract

**Background**: Observational studies from the USA have suggested that patients with low health literacy (HL) have higher health care costs and use an inefficient mix of health care services. To date, there were no studies from Europe that investigated the impact of HL on the use of the health system. The purpose of this study was to measure functional HL among persons having type 2 diabetes and to investigate the relationship between functional HL and health care costs and utilization. **Methods**: The study population were insured persons of the basic health insurance plan of the largest health insurer in Switzerland. Persons selected for participation had been reimbursed for diabetes medications in 2010–11, were aged 35–70 years and did not live in a long-term care institution. The level of functional HL was measured by one screening question. The following dependent variables were used: total costs, outpatient costs, inpatient costs, days admitted and number of physician visits attended. All multiple regression analyses were adjusted for age, gender, education, duration of diabetes, treatment with insulin (yes/no) and other chronic disease (yes/no). **Results**: High levels of functional HL were associated with lower total costs (*P* = 0.007), lower outpatient costs (*P* = 0.004) and less physician visits (*P* = 0.001). In the standard insurance plan with free access to all health professionals subgroup, the effects found were more pronounced. **Conclusions**: Persons with low functional HL need extra medical support, and therefore have higher health care costs.

## Introduction

The ability of citizens to take care of their own health and to navigate through increasingly complex health care systems has been recognized as a significant factor in health care costs and quality.^1^ Thus, health literacy (HL) has become an important research topic. The World Health Organization has defined HL as the “cognitive and social skills which determine the motivation and ability of individuals to gain access to, understand and use information in ways which promote and maintain good health.”^2^ Studies have shown that limited HL is associated with poorer health outcomes^3^^,^^4^ and lower use of preventive health services.^5–7^

For people suffering from diabetes, HL is even more important, as they have to be able to integrate health-related self-management activities in their daily life, such as following medication plans, respecting dietary intake modifications or being able to communicate with health care providers.^8^ To date, studies of the relationship between HL and diabetes control have provided mixed results. Some studies found associations between low HL and worse glycaemic control,^9^^,^^10^ more hypoglycaemic events^11^ and higher rates of retinopathy.^12^ Other studies did not confirm a relationship between glycaemic control and HL.^13^^,^^14^

However, different studies found associations between low HL and higher total health care spending,^15^ higher emergency department use,^16^ and higher inpatient costs.^7^^,^^17^ Eichler, et al.^18^ estimated the additional costs on the health system level associated with low HL in the range of 3 to 5% of the total health care costs per year. All these studies were based on data from the USA. Considering the important differences between the European and US living conditions and the health care systems, these findings cannot be generalized.

For example, in Switzerland basic health care insurance is compulsory for all citizens. They can choose between different insurers, and the insurers are obliged to accept every person in the basic health care insurance. Independent of the chosen insurer, the basic health care insurance covers the same medical costs for diagnostic and therapeutic procedures approved by law. Outpatient costs include all diagnostic and therapeutic procedures prescribed or conducted by approved physicians in the ambulatory setting. Inpatient costs include all diagnostic and therapeutic procedures undertaken in approved hospitals. Copayment of medical costs by the insured persons consists of a deductible (annual amount paid by insured persons towards the costs of medical benefits) and an excess (percentage of the costs invoiced annually for medical benefits over and above the amount of the deductible, but no more than the maximum amount per year specified by law). Insured persons can choose to pay higher annual deductibles or choose between different alternative insurance options with a restricted choice of service providers in return for reduced premiums.

For our study, we selected patients with type 2 diabetes because diabetes is one of the most common diseases, with 366 million people affected worldwide in 2011.^19^ In Switzerland, >300 000 people are suffering from diabetes, with ≈15 000 persons newly diagnosed per year.^20^ About 2.2% of the country's total health care expenditures are spent for the treatment of diabetes and its complications.^21^ In particular, persons with a low socioeconomic status suffer up to four times more frequently from diabetes than persons with a high socioeconomic status.^22^

Even though HL as a concept has been subject to constant changes and refinements, the development of measurements still lags behind.^23^ Although most definitions assume that there is more to HL than functional literacy, comprehensive tools that capture this multidimensionality are still lacking.^24^ The Test of Functional Health Literacy in Adults (TOFHLA)^25^ and the Rapid Estimate of Adult Literacy in Medicine (REALM)^26^ are two of the most commonly used measurement tools. Both test measure functional HL, where the TOFHLA aims at capturing patients’ reading comprehension and numeracy skills and REALM captures word recognition and pronunciation skills.

Another way to measure HL is via screening questions. Most of these questions ask about one’s ability to understand health-related materials and some have tried to capture the multidimensionality of the concept. Nevertheless, lack of external validity often poses a problem, and so far none of these measures have been considered to be the gold standard.^27^

The purpose of this study was to measure functional HL among persons having type 2 diabetes and to examine the relationship between HL and health care costs and utilization. To the best of our knowledge, this is the first study conducted in Europe, combining functional HL measurement of patients suffering from type 2 diabetes and the cost outcome data.

## Methods

### Study population

The study population were insured persons of the basic health insurance plan of the Helsana Group, the largest health insurer in Switzerland. Persons were eligible for the study if they had been reimbursed for diabetes medications (Anatomical Therapeutic Chemical classification system A10A or A10B) in 2010 and 2011, were not living in a long-term care institution and were aged between 35 and 70 years.

There are different official languages in different parts of Switzerland. As HL may interfere with the knowledge of the language, persons were eligible only if they had chosen the official language within the region where they were living as the correspondence language with the insurance company.

A randomized sample consisting of 7550 persons in the German-speaking part, 2926 persons in the French-speaking part and 1111 persons in the Italian-speaking part of Switzerland was contacted by letter. For enrolment they had to return a consent form by prepaid envelope to the University of Lugano, indicating whether they were suffering from type 2 diabetes mellitus.

### Interview data

Participants were interviewed by trained students of the University of Lugano in spring 2012. Telephone interviews were conducted in three official languages of the respective regions. Collected data included demographic information, information on health status and functional HL as measured by one screening item (More measures of HL have been included in the original questionnaire but for the purpose of this study it was decided to focus on the measure for functional HL).

Because TOFHLA or REALM are not suitable for telephone interviews, we used one of the three screening items developed by Chew et al.^28^^,^^29^ All three items refer to functional HL, asking patients about their perceived competence to understand health-related information material. The studies of Chew et al. showed that the item “How often do you have problems learning about your medical condition because of difficulty understanding written information?” had an AUROC of 0.76 (95% CI = 0.62–0.90) for identifying persons with inadequate HL and an AUROC of 0.60 (95% CI = 0.0.51–0.69) for identifying persons with inadequate or marginal HL. The authors indicated in their study that the combination of all three items would not lead to any significant change in detecting HL levels.^29^ Therefore, we chose only one of the three items because this one was the most applicable to the Swiss health care context. As the translation of the original question could be misinterpreted, we used a slightly adapted version: *“*When you get written information on a medical treatment or your medical condition, how often do you have problems understanding what it is telling you?*”*

The study protocol was approved by the appropriate regional ethical committee ‘Comitato etico cantonale, Ticino’.

### Insurance data

We retrieved all costs and utilization data for2010 and 2011 from the insurer’s administrative database including all copayments (deductible and excess). We distinguished three categories of health insurance plans. The standard insurance plan included free access to all physicians, in the telephone counselling plan the insured person had to call a medical call centre before going to a physician and in the gatekeeper-model the insured person had to first go to a selected physician.

### Statistical analysis

We corrected the non-normal distribution of costs and utilization data by using log transformation. To identify differences between participants and non-participants in health care costs and utilization, we used Levene’s test for homogeneity of variance and the independent *t*-test for equality of means.

Multiple regression analyses were conducted to analyse the relationship between the level of functional HL and health care costs and utilization. As dependent variables for health care costs, we used total costs, outpatient costs and inpatient costs. Based on the data available, the utilization of the health care system was assessed on the basis of the number of days admitted in hospital and the number of physician visits attended. Levels of HL were used as independent dummy variables comparing the different levels of functional HL with the group having the highest level of functional HL. All regression models included the covariates age, gender, duration of diabetes, treatment with insulin, other chronic condition and education. To control for influences of the chosen health insurance plan, we performed a subgroup analyses of persons choosing the standard insurance plan. All statistical analyses were performed using SPSS version 19.

## Results

### Sample characteristics

**Figure 1:**
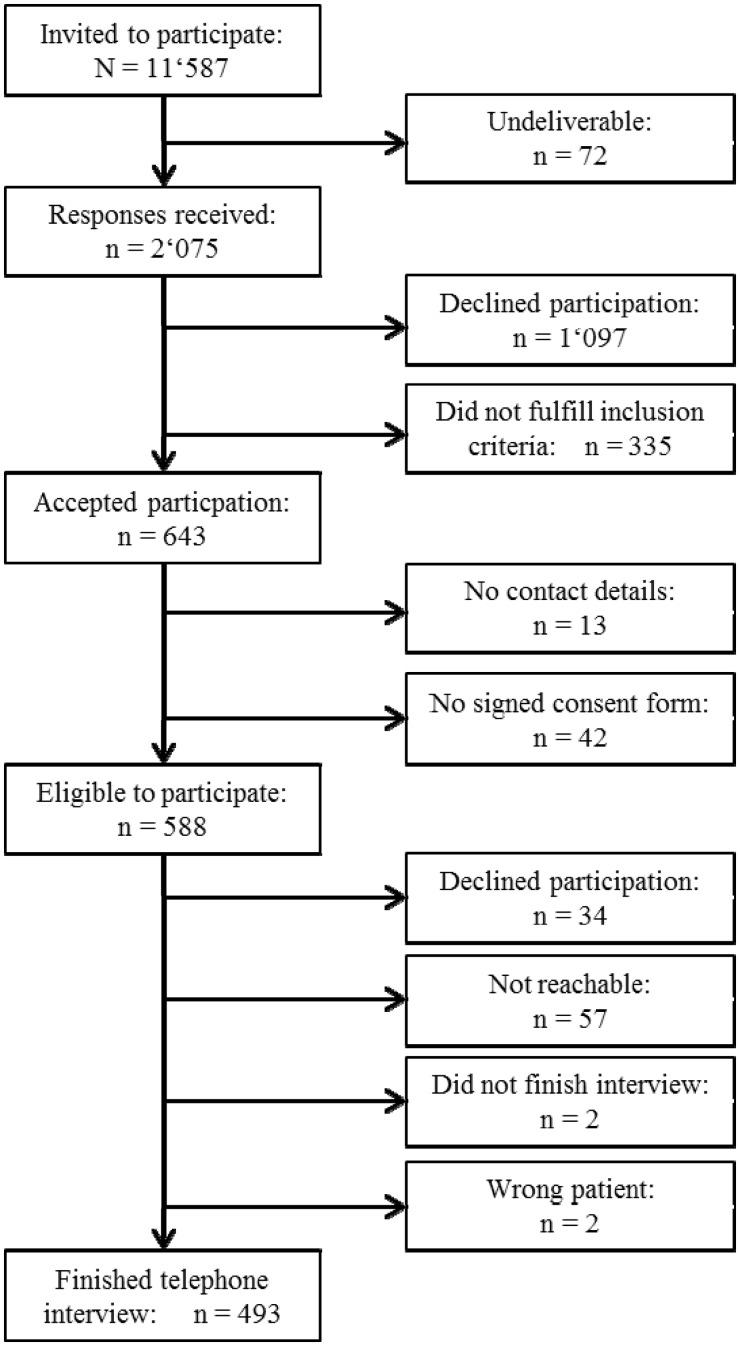
Enrolment process

From the 11 587 persons contacted, we received 2075 response cards, of which 1097 declined participation and 335 persons did not fulfil the inclusion criteria because they did not have type 2 diabetes. Of the 588 eligible persons, 34 later declined participation, 57 were not reachable by phone, 2 did not finish the interview and in 2 cases the interviewed persons were not the selected persons. Finally, interview data were obtained from 493 persons (figure 1).

We found that males, or those aged >65 years, or those living in the German-speaking region of Switzerland, or those having chosen a higher cost participation or a gatekeeper-model as insurance plan were more likely to participate (table 1).

**Table 1:** Study population characteristics

2011	Participants		Non-participants		Total		χ^2^	*P*-value
	*n*	%	*N*	%	*N*	%		
Gender								
Male	332	67.3	6690	60.3	7022	60.6	9.798	**0.002**
Female	161	32.7	4404	39.0	4565	39.4		
Age								
35–44	8	1.6	743	6.7	751	6.5	123.231	**<0.001**
45–54	42	8.5	2258	20.4	2300	19.8		
55–64	189	38.3	4743	42.8	4932	42.6		
65–70	254	51.5	3350	30.2	3604	31.1		
Language								
German	391	79.3	7159	64.5	7550	65.2	45.42	**<0.001**
French	74	15.0	2852	25.7	2926	25.3		
Italian	28	5.7	1083	9.8	1111	9.6		
Annual deductible								
≤ € 250	350	71.0	7613	68.6	7963	68.7	11.158	**0.004**
= € 417	105	21.3	2917	26.3	3022	26.1		
≥ € 833	38	7.7	564	5.1	602	5.2		
Insurance plan								
Standard	274	55.6	7738	69.7	8012	69.1	45.756	**<0.001**
Telephone counselling	58	11.8	992	8.9	1050	9.1		
Gatekeeper model	161	32.7	2364	21.3	2525	21.8		
Total	493	100	11 094	100.0	11 587	100		

Of the 493 participants, 441 (89%) had been diagnosed with type 2 diabetes >5 years ago, and 164 participants (33%) were treated with insulin. In addition, 227 participants (46%) had other chronic disease. Twelve persons reported having suffered from diabetes for 1 year or less, but according to the insurance claims data all participants received medications against diabetes for at least 2 years.

### Functional HL

Half of the participants declared never having problems in understanding written information related to their medical condition. In contrast, 7.3% of the participants often or always had problems understanding written information (table 2).

**Table 2:** Results of HL screening question

	*n*	%
Always	12	2.4
Often	24	4.9
Sometimes	78	15.8
Occasionally	129	26.2
Never	250	50.7
Total	493	100

“When you get written information on a medical treatment or your medical condition, how often do you have problems understanding what it is telling you?”.

### Health care costs and utilization

For health care costs and utilization, the Levene’s test was non-significant in all categories, confirming the homogeneity of variances in participants and non-participants.

In 2011, participants on average caused significantly higher total costs [*GM* = geometric mean (*GM*) = € 5155, *SE* = 1.05 vs. *GM* = € 4393, *SE* = 1.01; *t* (11 585) = 3.10, *P* = 0.002], higher outpatient costs (*GM* = € 4137, *SE* = 1.04 vs. *GM* = € 3669, *SE* = 1.01; *t* (11 585) = 3.06, *P* = 0.002) and more physician visits (*GM* = 24, *SE* = 1.04 vs. *GM* = 20, *SE* = 1.01; *t* (11 199) = 5.10, *P* < .001) than non-participants. These differences were confirmed by the data of 2010.

### Multiple regression analyses

Multiple regression analyses were conducted to evaluate the association between HL and different health care costs and utilization variables. The results of the regressions suggested that persons with lower levels of HL caused higher total costs, higher outpatient costs and had more physician visits (table 3). In the subgroup analysis of patients who chose the standard insurance plan with free access to all health professionals, the effects found were more pronounced (table 4).

**Table 3:** Regression coefficients quantifying associations between HL screening question and health care costs and utilization

Year 2011 (*n* = 492)									Year 2010 (*n* = 492)								
Dependent variables	Predictive variable	*R^2^*	*B*	*SE*	*Beta*	*P*	95.0% *CI* for B		Dependent variables	Predictive variable	*R^2^*	*B*	*SE*	*Beta*	*P*	95.0% *CI* for B	
Total costs	Occasionally	0.152	−0.050	0.115	−0.020	0.663	−0.275	0.175	Total costs	Occasionally	0.152	0.068	0.113	0.027	0.549	−0.154	0.289
	Sometimes		0.104	0.138	0.034	0.449	−0.166	0.375		Sometimes		0.243	0.135	0.081	0.073	−0.023	0.508
	Often		0.342	0.225	0.066	0.130	−0.101	0.784		Often		0.342	0.221	0.067	0.123	−0.093	0.777
	Always		0.849	0.312	0.118	**0.007**	0.236	1.463		Always		0.626	0.307	0.088	**0.042**	0.023	1.228
Outpatient costs	Occasionally	0.226	−0.078	0.086	−0.039	0.362	−0.247	0.091	Outpatient costs	Occasionally	0.176	0.020	0.088	0.010	0.816	−0.152	0.193
	Sometimes		0.038	0.103	0.016	0.715	−0.165	0.241		Sometimes		0.109	0.106	0.046	0.304	−0.099	0.316
	Often		0.256	0.169	0.063	0.130	−0.076	0.588		Often		0.256	0.173	0.064	0.139	−0.083	0.596
	Always		0.673	0.234	0.119	**0.0**04	0.213	1.133		Always		0.544	0.240	0.097	**0.024**	0.073	1.015
Inpatient costs	Occasionally	0.149	0.100	0.223	0.049	0.656	−0.342	0.541	Inpatient costs	Occasionally	0.148	0.233	0.241	0.108	0.336	−0.246	0.712
	Sometimes		0.158	0.281	0.064	0.574	−0.399	0.715		Sometimes		0.072	0.275	0.031	0.794	−0.475	0.619
	Often		0.754	0.419	0.186	0.075	−0.077	1.585		Often		0.766	0.443	0.187	0.087	−0.114	1.647
	Always		0.466	0.425	0.115	0.276	−0.378	1.310		Always		0.152	0.487	0.034	0.756	−0.817	1.120
Days admitted	Occasionally	0.094	0.025	0.257	0.011	0.921	−0.485	0.535	Days admitted	Occasionally	0.192	0.291	0.256	0.127	0.258	−0.217	0.799
	Sometimes		−0.051	0.324	−0.018	0.875	−0.694	0.592		Sometimes		0.130	0.298	0.051	0.664	−0.463	0.723
	Often		0.177	0.483	0.039	0.715	−0.782	1.136		Often		1.025	0.471	0.236	0.032	0.089	1.961
	Always		0.655	0.490	0.145	0.185	−0.318	1.629		Always		0.311	0.514	0.065	0.547	−0.711	1.332
Physician visits	Occasionally	0.088	−0.072	0.084	−0.040	0.394	−0.237	0.093	Physician visits	Occasionally	0.090	0.043	0.083	0.024	0.609	−0.121	0.206
	Sometimes		0.179	0.100	0.084	0.075	−0.018	0.377		Sometimes		0.234	0.100	0.111	**0.019**	0.038	0.430
	Often		0.123	0.164	0.034	0.454	−0.200	0.446		Often		0.273	0.163	0.076	0.094	−0.047	0.593
	Always		0.786	0.228	0.156	**0.001**	0.339	1.234		Always		0.751	0.225	0.150	**0.001**	0.308	1.194

Note: All regressions included the covariates age, gender, education, duration of diabetes, treatment with insulin (yes/no) and other chronic disease (yes/no).

**Table 4:** Regression coefficients of the subgroup analysis ‘Standard insurance plan’ quantifying associations between HL screening question and health care costs and utilization

Year 2011 (*n* = 274)									Year 2010 (*n* = 292)								
Dependent variables	Predictive variable	*R^2^*	*B*	*SE*	*Beta*	*P*	95.0% *CI* for B		Dependent variables	Predictive variable	*R^2^*	*B*	*SE*	*Beta*	*P*	95.0% *CI* for B	
Total costs	Occasionally	0.200	0.085	0.151	0.034	0.574	−0.213	0.382	Total costs	Occasionally	0.174	0.179	0.150	0.070	0.234	−0.117	0.475
	Sometimes		0.272	0.184	0.088	0.142	−0.091	0.634		Sometimes		0.152	0.176	0.050	0.388	−0.195	0.499
	Often		0.664	0.297	0.130	**0.026**	0.080	1.248		Often		0.342	0.266	0.074	0.199	−0.181	0.864
	Always		1.483	0.424	0.200	**0.001**	0.648	2.319		Always		1.260	0.430	0.165	**0.004**	0.414	2.105
Outpatient costs	Occasionally	0.276	−0.004	0.113	−0.002	0.971	−0.226	0.218	Outpatient costs	Occasionally	0.191	0.064	0.116	0.032	0.583	−0.164	0.291
	Sometimes		0.120	0.138	0.049	0.383	−0.151	0.391		Sometimes		−0.008	0.136	−0.003	0.956	−0.274	0.259
	Often		0.342	0.222	0.085	0.124	−0.095	0.778		Often		0.317	0.204	0.088	0.122	−0.085	0.719
	Always		1.219	0.317	0.210	**0.001**	0.595	1.843		Always		1.073	0.331	0.181	**0.001**	0.422	1.723
Inpatient costs	Occasionally	0.233	0.147	0.273	0.072	0.591	−0.399	0.693	Inpatient costs	Occasionally	0.345	0.457	0.283	0.208	0.113	−0.111	1.025
	Sometimes		0.139	0.330	0.059	0.675	−0.522	0.800		Sometimes		−0.319	0.343	−0.130	0.356	−1.007	0.368
	Often		0.907	0.462	0.251	0.054	−0.018	1.831		Often		1.053	0.596	0.223	0.083	−0.144	2.250
	Always		0.262	0.473	0.073	0.582	−0.684	1.208		Always		0.269	0.546	0.065	0.625	−0.827	1.364
Days admitted	Occasionally	0.162	0.069	0.312	0.031	0.826	−0.556	0.694	Days admitted	Occasionally	0.281	0.467	0.340	0.193	0.177	−0.217	1.151
	Sometimes		−0.241	0.378	−0.093	0.526	−0.998	0.516		Sometimes		−0.135	0.415	−0.048	0.746	−0.969	0.698
	Often		0.077	0.529	0.019	0.885	−0.982	1.135		Often		1.193	0.709	0.231	0.098	−0.230	2.617
	Always		0.327	0.541	0.083	0.547	−0.755	1.410		Always		0.426	0.648	0.095	0.514	−0.877	10.729
Physician visits	Occasionally	0.143	−0.061	0.117	−0.032	0.606	−0.292	0.171	Physician visits	Occasionally	0.116	0.049	0.111	0.027	0.657	−0.169	0.267
	Sometimes		0.301	0.142	0.131	**0.035**	0.021	0.581		Sometimes		0.211	0.129	0.099	0.103	−0.043	0.464
	Often		−0.047	0.229	−0.012	0.838	−0.497	0.403		Often		0.238	0.194	0.073	0.222	−0.145	0.620
	Always		1.202	0.327	0.220	**0.001**	0.559	1.846		Always		1.206	0.314	0.226	**0.001**	0.588	1.825

Note: All regressions included the covariates age, gender, education, duration of diabetes, treatment with insulin (yes/no) and other chronic disease (yes/no).

## Discussion

The study demonstrated that patients with type 2 diabetes with low functional HL had higher total and outpatient costs. This is also reflected by the fact that patients with lower HL levels visited their physicians more frequently than patients with higher levels of HL, as visiting the physician is reflected in both total and outpatient costs. Functional HL was operationalized by self-reported competence to understand written information about their medical condition.

Some further observations substantiate this finding. Inpatient costs and the number of days admitted to a hospital also tended to be higher in low-HL patients, but the difference did not reach a significant level. Nevertheless, the fact that with each decreasing level of HL, the association became stronger (increasing regression betas), further supports the general finding. Improving the evidence beyond what other studies show, we were able to demonstrate that the negative relationship between levels of functional HL and health care costs and health system utilization was stable over the 2 years.

Our findings of a relationship between HL and outpatient costs and the number of physician visits are in contrast to studies conducted in the USA, where office visit rates were shown to be similar across the range of HL scores.^15^ Further, studies from the USA showed that lower HL was associated with higher emergency department and inpatient admission costs,^7^^,^^15^ therby suggesting that low-HL patients there may use an inefficient mix of health services. Our results do not suggest similar findings for Switzerland. One explanation could be the general low number of hospital days in our sample. Another explanation could be that, in contrast to the USA, the fast and easy access to physicians in private practices in Switzerland diminishes the number of hospital admissions and inpatient costs. This would also explain the greater effect we found on the physician visits and the outpatient costs. That people with low HL visit their physician more often can also be traced back to their worse glycaemic control and/or more comorbidities and the higher need for medical support caused by this.

In the subgroup of persons having chosen the standard insurance plan without telephone counselling or a gatekeeper, the effects were even more pronounced. This might be due to the fact that persons with low HL would need these services more, and not having them, their insecurity in the choice of the appropriate health care provider results in navigating through turning to the health care system more frequently.

Our sample showed a low variance of HL levels, which made it more difficult to discern significant differences. Most studies identified at least 20% of patients with low HL.^30^ In our sample, only 7.3% persons were considered to have inadequate HL. But according to the Adult Literacy and Life Skills Survey^31^, at least 40% of the Swiss population have low levels of general problem-solving, literacy and numeracy skills. As HL is correlated with education, we assume that persons with low HL were underrepresented in our sample due to participation bias. Despite this constraint, the association between low HL and health care costs and utilization remained significant. The likely underrepresentation of persons with low HL makes us conclude that our study underestimates the true effect of low HL on health care costs and health care system utilization in Switzerland.

There are several limitations to this study. First, as most of the participants showed a high level of HL, it is possible that the chosen item for its measurement was not sufficiently accurate. However, more established instruments, such as the S-TOFHLA or the REALM, could not be used for telephone interviews. The next best choice was a validated item from Chew et al.^28^^,^^29^

Second, although the presence of another chronic disease was integrated as a covariate in all regression models, we had no medical data to confirm the self-reported comorbidities. Low HL could result in underreporting comorbidities, and therefore lead to overestimation of the effect of low HL on health care costs and use. On the other hand, more comorbidities could mediate the relationship between low HL and higher health care costs and utilization.^30^ In this case, controlling for comorbidities could have led to an underestimation.

Third, even if our study population was representative for patients with type 2 diabetes in Switzerland, participants and non-participants were significantly different with regard to age, gender, chosen insurance plan, total costs, outpatient costs and the number of physician visits. Therefore, the findings of our study cannot be considered to be truly representative of patients with type 2 diabetes in Switzerland. Furthermore, due to the low number of participants from the French- and Italian-speaking regions of Switzerland, our results reflect mostly the situation in the German-speaking region of Switzerland. Therefore, our findings cannot be applied to the two smaller language regions with the same reliability as to the German-speaking region. However, our study suggests that even in a country with compulsory health insurance coverage and a renowned health care system, HL influences the use of the health care system and the health care costs. Finally, the observational design does not allow defining if any association found was causal.

Besides the limitations of this study, several questions remain unanswered. We used the understanding of written information as a proxy for low functional HL. But the concept of HL contains far more than reading and numeracy skills.^32^^,^^33^ Further studies are needed to better understand the broader concept of HL and to improve HL measurements. As this is the first study conducted in Europe to evaluate the relationship between functional HL and health care costs and utilization, more studies are needed to investigate the impact of low HL on an individual and societal level.

## Funding

The Helsana Insurance Company financed the production and shipment of the invitation mailings to the selected persons and the interviews performed by University of Lugano.

*Conflict of interest*. All authors have completed the Unified Competing Interest form at www.icmje.org/coi_disclosure.pdf (available on request from the corresponding author) and declare that (i) J.F. and R.R. are employed by the Helsana Group, but the sponsor had no role in the planning, conducting or submission of this manuscript; (ii) S.M. and P.J.S. have no relationships with the Helsana Group in the previous 3 years; (iii) their spouses, partners or children have no financial relationships that may be relevant to the submitted work; and (iv) J.F., R.R., S.M. and P.J.S. have no non-financial interests that may be relevant to the submitted work. Helsana Group shall have no liability to any third party with respect to the contents of this article.

**Key points**

- Observational studies from the USA have suggested that patients with low HL have higher health care costs and use an inefficient mix of health care services.
- Our study suggests that even in a European country with compulsory health insurance, HL influences the use of the health care system and the health care costs.
- Persons with low HL need more medical support, and therefore have higher health care costs.
